# Paraganglioma of the Urinary Bladder: A Case Report on a Rare and Unexpected Tumor Location

**DOI:** 10.7759/cureus.41998

**Published:** 2023-07-17

**Authors:** Zisis Kratiras, Aris Kaltsas, Nektarios Koufopoulos, Konstantinos Adamos, Michail Chrisofos

**Affiliations:** 1 Third Department of Urology, School of Medicine, Attikon University Hospital, University of Athens, Athens, GRC; 2 Department of Urology, Faculty of Medicine, School of Health Sciences, University of Ioannina, Ioannina, GRC; 3 Second Department of Pathology, School of Medicine, Attikon University Hospital, University of Athens, Athens, GRC

**Keywords:** treatment, diagnosis, transurethral resection of bladder tumor, immunohistochemistry, urinary bladder, paraganglioma

## Abstract

Paraganglioma of the urinary bladder is an exceptionally rare tumor. It originates from chromaffin cells, which are responsible for producing catecholamines. We report a unique case of a 74-year-old woman diagnosed with nonfunctional bladder paraganglioma, who presented with macroscopic hematuria and right-sided renal colic but lacked the usual symptoms associated with catecholamine excess. This case highlights the diagnostic challenges of nonfunctional variants of paraganglioma due to their histological similarity to urothelial carcinomas. It underscores the importance of a thorough histological examination and the need for a multidisciplinary approach to establish a diagnosis and determine the optimal treatment strategy. Our case contributes to the sparse literature on this rare condition, and it aims to enhance clinicians' awareness and understanding of urinary bladder paragangliomas.

## Introduction

Paragangliomas (PGLs) are rare neoplasms derived from the catecholamine-producing chromaffin cells of the sympathetic nervous system [1]. Their presence across the body leads to the possibility of PGL occurrence in unexpected sites, one of which is the urinary bladder. Paraganglioma of the urinary bladder, which contributes to less than 0.1% of bladder tumors, remains a topic of enduring interest in urology due to its rarity [2].

Clinically, bladder PGLs can manifest with a spectrum of symptoms, including, but not limited to, palpitations, sweating, headaches, paroxysmal hypertension during micturition, and hematuria [3]. Intriguingly, these symptoms are often attributable to excessive catecholamine secretion. In some cases, patients can experience hypertensive crises during surgical procedures, further complicating their management [4].

The diagnostic journey for bladder PGLs is often fraught with challenges. One of the major hurdles lies in differentiating bladder paraganglioma from urothelial cancer, a far more common bladder malignancy. This distinction is of utmost importance, considering that these two entities call for different therapeutic approaches [5]. Histological examination, therefore, becomes a cornerstone in establishing an accurate diagnosis [6].

Managing bladder paraganglioma necessitates an individualized approach, primarily involving surgical intervention such as transurethral resection or cystectomy [2]. Factors influencing the choice of treatment include the extent of the tumor and the presence of metastasis [7]. It is important to note that patients with localized tumors generally have a favorable prognosis and may be managed with less aggressive treatment modalities. However, those presenting with metastatic disease usually face reduced survival rates, even with aggressive intervention [7].

The literature features several case reports detailing the unique presentations and management of bladder paraganglioma. Cases have described young and middle-aged females presenting with a wide array of symptoms, from accelerated hypertension and visual disturbances to abdominal pain and intermittent hematuria [8,9]. These individual case presentations underscore the importance of maintaining a high degree of suspicion for bladder paraganglioma when encountering patients with relevant symptoms.

Through the presentation of this unusual case, we aim to add to the existing knowledge and inspire heightened awareness among clinicians about this rare condition. As with many rare entities, its proper recognition and management can make a significant difference in patient outcomes.

## Case presentation

**Figure 1:**
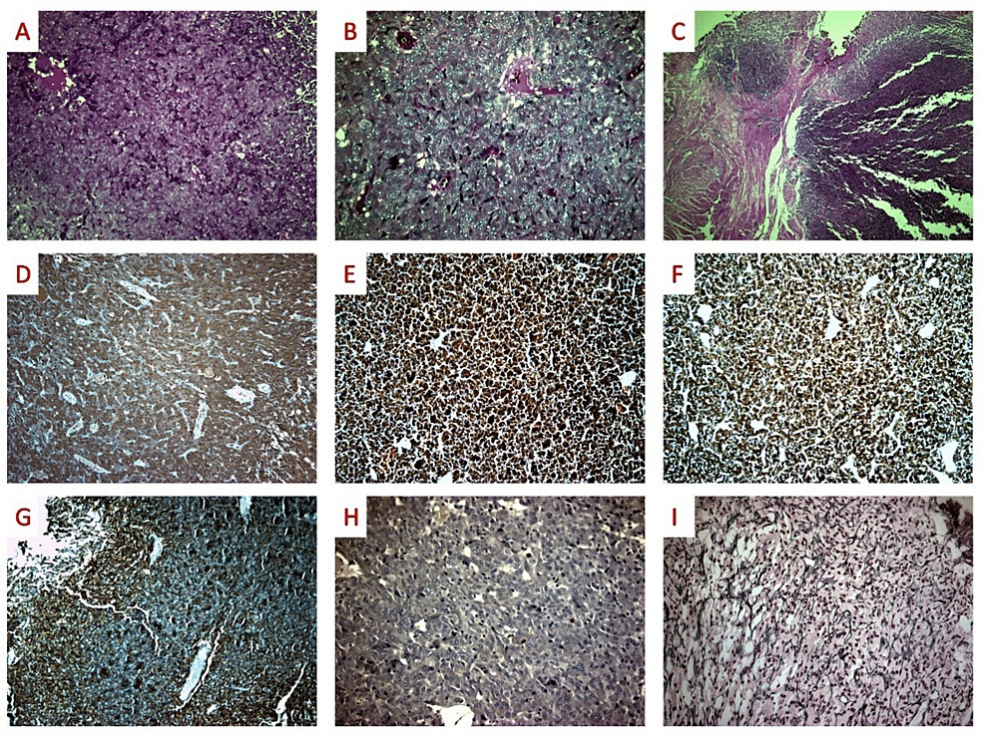
Histopathologic characteristics of nonfunctional bladder paraganglioma. Histopathologic and immunohistochemical findings from the transurethral resection specimen of a nonfunctional bladder paraganglioma. (A and B) The tumor had a nested pattern of uniform nuclei with smooth chromatin amphophilic granular cytoplasm without necrosis atypia or mitotic figures (H&E, ×100; H&E ×200); (C) There was the involvement of the muscularis propria without eliciting a desmoplastic reaction (H&E, ×40). (D-F) On immunohistochemical examination, the cytoplasm of the tumor cells stained for S100 (S100 ×100), chromogranin (×100), and synaptophysin (×100). (G) CD56 showed strong diffuse membranous staining (CD56 ×100); (H) Ki67 stained around 1% of tumor nuclei (Ki67 ×200). (I) Histochemical reticulin stain highlighted the nesting pattern (reticulin ×200). H&E, hematoxylin and eosin

A 74-year-old woman with a medical history of arterial hypertension, diabetes mellitus, and depression presented to the emergency department with macroscopic hematuria and right-sided renal colic. Her clinical examination, including the digital rectal and bimanual examination, was unremarkable. Her blood results were within the normal range at admission, with a fasting capillary blood glucose of 105 mg/dL, creatinine at 0.9 mg/dL, urea at 79.7 mg/dL, hemoglobin at 12.4 g/dL, and a white blood cell count of 6940 K/μL. An initial ultrasound scan revealed pathological exophytic tissue at the dome and the upper right lateral wall of the bladder, with no notable findings in the kidneys. Given these findings, a bladder neoplasm was suspected, and the patient underwent transurethral resection of the bladder tumor the following day. During the procedure, the tumor was identified at the dome of the bladder, extending to the right lateral wall, close to the right ureteral orifice. A complete transurethral resection of the tumor was performed. The excised tumor, estimated at a volume of approximately 3 mL, was a well-circumscribed, yellow-brown solid mass invading the muscular and subserosal layers of the bladder. Microscopic examination revealed a neoplasm consisting of tumor cells with amphophilic granular cytoplasm, uniform nuclei with smooth chromatin, organized in nests (a zellballen pattern), separated by prominent fibrovascular stroma, involving the muscularis propria without desmoplastic reaction (Figures 1A-1C). Necrosis, giant multinucleated or bizarre cells, and mitotic figures were not identified. Immunohistochemical findings showed positive staining for S100 (Figure 1D), chromogranin A (Figure 1E), synaptophysin (Figure 1F), and CD56 (Figure 1G), while Ki67 proliferative index marker stained around 1% of tumor nuclei (Figure 1H). Epithelial and melanocytic markers (CKAE1/AE3, CK8/18, CK7, CK20, HMB45, and Melan A) were uniformly negative. A histochemical reticulin stain highlighted the nesting pattern (Figure 1I). Based on the above findings, we diagnosed this tumor as consistent with a bladder PGL. The patient had an unremarkable postoperative recovery without any complications and was discharged from the hospital on the third postoperative day. Following the pathological diagnosis, the patient was referred to the endocrinology department for further evaluation and follow-up. She had a repeat flexible cystoscopy in three and six months following her transurethral resection of bladder tumor (TURBT) that revealed no obvious recurrence.

## Discussion

Extra-adrenal paragangliomas are neuroendocrine neoplasms predominantly arising from chromaffin cells of the autonomic nervous system. Given that chromaffin cells are located in the detrusor muscle of the bladder wall, paragangliomas in the urinary bladder are rare [8]. Due to its rarity and the lack of specific symptoms, preoperative diagnosis can be challenging [10]. Patients with bladder paraganglioma may present with a variety of symptoms, including palpitations, paroxysmal hypertension, sweating, headaches, and hematuria [8]. These symptoms are often due to catecholamine excess. In some cases, patients may experience hypertensive crises during surgical procedures. Therefore, it is crucial to consider paraganglioma as a differential diagnosis in patients with relevant symptoms. However, our patient's case presented complexities beyond the norm as hematuria was her first symptom and there was no evidence of catecholamine release. In addition, follow-up examinations of CT revealed no evidence of new tumor masses elsewhere in the body.

Accurate diagnosis of bladder paraganglioma is essential, as treatment options differ from those for urothelial cancer. Histological examination is crucial for confirming the diagnosis. Several factors contribute to the frequent misdiagnosis of urinary bladder paragangliomas (UBPGL) as urothelial carcinoma. These include frequent involvement of the muscularis propria, histomorphology that mimics urothelial carcinoma, the omission of UBPGL in differential diagnoses when evaluating bladder tumors, and the small minority of patients with identifiable catecholamine-associated symptoms [5]. This aspect is vital as, despite only a small percentage of bladder PGL patients presenting these symptoms, 83% of bladder PGLs are hormonally active, thereby contributing to the complexity of diagnosis [6].

Surgical intervention, such as transurethral resection or cystectomy, is the mainstay of treatment for localized tumors. Misdiagnosing UBPGL as urothelial carcinoma can lead to severe consequences as these conditions require different treatments and have different prognoses. Treatment of urothelial carcinoma depends on the stage of the disease; intravesical Bacillus Calmette-Guerin (BCG), surveillance, and repeated transurethral excision of the bladder tumor (reTURBT) are commonly used to treat non-muscle-invasive carcinomas. In cases of locally advanced paraganglioma, partial cystectomy and extended pelvic lymph node dissection may be performed [7]. The prognosis for patients with localized tumors is generally favorable, while those with metastatic disease have a reduced survival rate despite aggressive treatment. External beam radiation and stereotactic radiosurgery have been investigated as treatment modalities for paragangliomas, particularly in cases where surgical intervention is not feasible [11]. In a study investigating long-term results of external-beam radiotherapy and stereotactic radiosurgery for paragangliomas, a 10-year tumor control rate of 92% was reported [11]. However, for PGL, TURBT or partial cystectomy, where the tumor is completely removed even if it has invaded the muscle, is usually the most successful procedure [12,13].

Histologically, UBPGLs exhibit a cell-ball pattern characterized by nests of tumor cells separated by narrow fibrovascular septa. If not detected immediately, a thorough search is required. Large, epithelioid tumor cells are present with stable monomorphic nuclei and an abundance of eosinophilic/amphophilic and granular cytoplasm. Additionally acceptable are rare, peculiar nuclear atypia [5]. These histologic features may help distinguish UBPGL from urothelial carcinoma and other differential diagnoses, along with a distinct immunohistochemical profile [14-16].

UBPGLs are genetically diverse and typically have a deletion of 1p, 3q, and 22q. Up to 30% of paragangliomas are thought to be familial and associated with neurofibromatosis type 1 (NF1), multiple endocrine neoplasia type 2 (MEN 2), von Hippel-Lindau syndrome (VHL), and familial paraganglioma-pheochromocytoma syndromes (SDH gene). A mutation in SDH increases the likelihood of extra-adrenal paraganglioma developing into cancer [13]. Our patient did not undergo any genetic syndromic study; however, there was no paraganglioma syndrome complex family history.

The rarity of bladder paraganglioma highlights the importance of reporting and documenting cases to enhance our understanding of this rare tumor and improve patient outcomes. Case reports have played a significant role in describing unique presentations, diagnostic challenges, and management strategies for bladder paraganglioma [2,7]. These reports contribute to the existing literature and provide valuable insights into the clinical characteristics and treatment options for this rare tumor. 

## Conclusions

The distinction between urinary bladder paragangliomas, particularly their nonfunctional variants, and urothelial carcinomas can present significant challenges. Nevertheless, biochemical tests of urine/plasma catecholamines should be considered if clinical suspicion arises. Additionally, imaging techniques such as CT, MRI, or metaiodobenzylguanidine (MIBG) scintigraphy can assist in confirming the diagnosis, identifying the metastatic disease, and guiding the surgical plan. Pathological examination remains crucial for a definitive diagnosis. Despite the rarity of bladder paragangliomas, the need persists for the establishment of a standardized surgical approach. Further research, including more detailed case reports, is essential to deepen our understanding of this rare tumor and improve patient outcomes.
